# Effectiveness of the home-based alcohol prevention program "In control: No alcohol!": study protocol of a randomized controlled trial

**DOI:** 10.1186/1471-2458-11-622

**Published:** 2011-08-04

**Authors:** Suzanne HW Mares, Haske van der Vorst, Anna Lichtwarck-Aschoff, Ingrid Schulten, Jacqueline EE Verdurmen, Roy Otten, Rutger CME Engels

**Affiliations:** 1 Behavioural Science Institue, Radboud University Nijmegen, The Netherlands; 2 Trimbos Institute (Netherlands Institute of Mental Health and Addiction), Utrecht, The Netherlands

## Abstract

**Background:**

In the Netherlands, children start to drink at an early age; of the Dutch 12-year olds, 40% reports lifetime alcohol use, while 9.7% reports last-month drinking. Starting to drink at an early age puts youth at risk of developing several alcohol-related problems later in life. Recently, a home-based prevention program called "In control: No alcohol!" was developed to delay the age of alcohol onset in children. The main aim of this project is to conduct a Randomized Controlled Trial (RCT) to evaluate the effectiveness of the program.

**Methods/Design:**

The prevention program will be tested with an RCT among mothers and their 6 grade primary school children (11-12 years old), randomly assigned to the prevention or control condition. The program consists of five printed magazines and an activity book designed to improve parental alcohol-specific socialization. Parent-child dyads in the control group receive a factsheet information brochure, which is the standard alcohol brochure of the Trimbos Institute (the Netherlands Institute for Mental Health and Addiction).

Outcome measures are initiation of alcohol use (have been drinking at least one glass of alcohol), alcohol-specific parenting, susceptibility to drinking alcohol, alcohol expectancies, self-efficacy, and frequency and intensity of child alcohol use. Questionnaires will be administered online on secured Internet webpages, with personal login codes for both mothers and children. Mothers and children in both the experimental and control condition will be surveyed at baseline and after 6, 12, and 18 months (follow-ups).

**Discussion:**

The present study protocol presents the design of an RCT evaluating the effectiveness of the home-based "In control: No alcohol!" program for 6 grade primary school children (11-12 years old). It is hypothesized that children in the prevention condition will be less likely to have their first glass of alcohol, compared to the control condition. When the prevention appears to be effective, it can easily and relatively quickly be implemented as a standard alcohol prevention program on a large scale.

**Trial registration:**

Nederlands Trial Register NTR2564

## Background

Adolescence is characterized by a strong increase in alcohol use: In 2009, approximately 40% of all Dutch 12-year olds reported lifetime alcohol use, which increases to 70% among 14-year olds and 85% among 16-year olds [1]. Of the Dutch 12-year olds, 9.7% even reported to have been drinking in the last month [1]. Starting to drink at an early age puts youth at serious risk of developing many alcohol-related problems, such as heavy episodic drinking, alcoholism, and cognitive impairments (e.g., [2,3]). These consequences of early onset of alcohol use stress the need to postpone the age of onset. Most school-based alcohol prevention in the Netherlands is conducted at the secondary educational level among 12-15-year olds, while at this age many children have already started to experiment with alcohol. Since many Dutch youth start to drink in early adolescence, prevention programs targeting elementary schoolchildren are needed. However, theory-driven alcohol prevention programs for elementary schoolchildren are lacking.

Socialization theory [4] posits that parents are the main socializing agents in their children's development, especially when it comes to health issues, which has been supported by a wide range of studies [5,6]. Recent studies in the Netherlands showed that through e.g., setting strict rules about alcohol, communicating constructively about alcohol issues, and monitoring daily activities, parents can delay the onset of alcohol use [7-10]. Another reason why parents are important in preventing adolescent alcohol use is that elementary schoolchildren live at home and are still very susceptible to their parents' influences, while peers become more important during mid-adolescence and in some domains parental influence declines [9,11]. Moreover, most children get their first glass of alcohol from their parents [12]. By making parents aware of their role in introducing alcohol to their child, the age of alcohol onset can be delayed (e.g., [8]).

Parental drinking affects adolescent alcohol use through norm-setting and modeling [9,13]. Further, drinking parents tend to engage less in alcohol-specific socialization practices [7,14], probably because they do not consider themselves being credible in prohibiting their children from drinking. However, alcohol-specific socialization strategies like setting rules, monitoring and communicating constructively are also effective when parents are (heavy) drinkers themselves (e.g., [7,15]). Therefore, it is important to empower this specific group of parents to enhance the confidence alcohol-drinking parents have in the effectiveness of their alcohol-specific parenting strategies. The current program addresses this issue by increasing parents' comfort level in communicating with their children about (their own) alcohol use.

Thus, although there is substantial empirical evidence that parents can prevent early onset of drinking by engaging in alcohol-specific parenting, no effective prevention program for parents and primary school children has been implemented in the Netherlands. The prevention program "In control: No alcohol!", which approach is based on a smoking prevention program called "Smoke-free Kids" [16], aims to fill this gap. It is a home-based program, which provides many opportunities to engage in structured interactions for the parents and children. Parents and children can go through the program on their own, when they have time, and are not obliged to engage in a complex, time-consuming program. A pilot study conducted over a period of 6 months has provided some insight into the effective components of the "In control: No alcohol!" program [17]. Specifically, exposure to the program increased the likelihood that mothers make a rule with their children about not drinking before a certain age, that mothers monitor their children, and for mothers that drink alcohol more than average, it increased the quality of alcohol-specific communication.

### Aim and hypotheses

The main aim of this project is to conduct a Randomized Controlled Trial (RCT) to evaluate a recently developed home-based alcohol prevention program, entitled "In control: No alcohol!" The program focuses on alcohol-specific parenting as a tool in delaying the age of alcohol onset in children. Onset of alcohol use is defined as the intake of the first glass of alcohol. With this RCT, including an experimental and a control group, we test whether fewer children have their first drink at an earlier age when included in the program. The second aim is to determine whether the program increases maternal use of several alcohol-specific parenting practices according to mothers and their offspring. The third aim is to test whether the prevention program differs between families varying on parental own drinking.

More specifically, we expect that a) a significant lower percentage of children who followed the program will have had their first glass of alcohol at the last follow-up compared to children who did not follow the program. b) mothers who followed the program are significantly more likely to engage in alcohol-specific parenting than mothers who did not follow the program. We expect that mothers involved in the program (as compared to controls) will set and keep stricter rules about alcohol, are more involved in constructive communication on alcohol-related topics, have more confidence in discussing alcohol matters, reduce children's access to alcohol beverages, make a rule with their children about not drinking before a certain age and are more likely to monitor children's activities. c) Above average drinking mothers that follow the program are more likely to increase their alcohol-specific parenting as compared to below average drinking mothers that follow the program.

## Methods/Design

### Study Design

**Figure 1:**
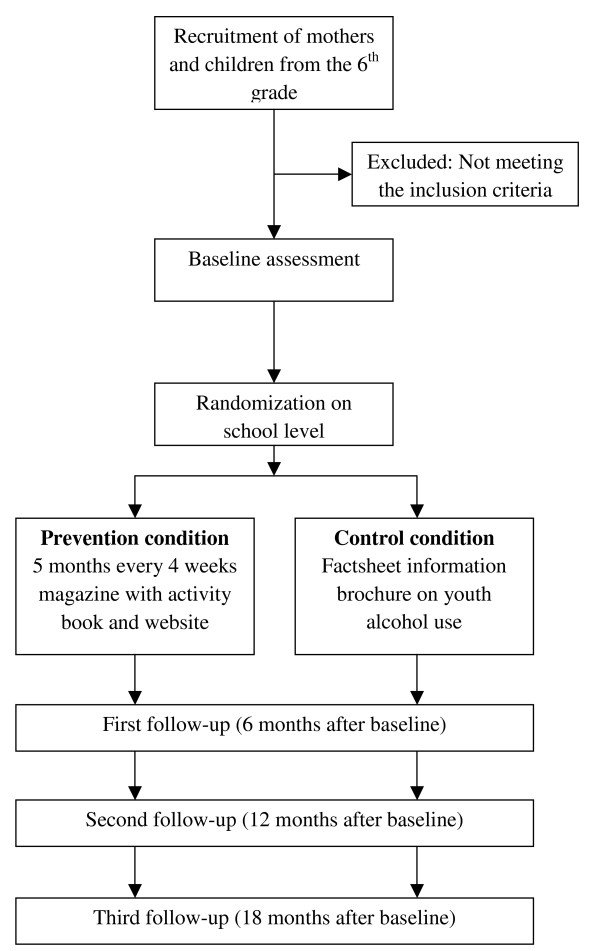
**Study Design**.

The prevention program "In control: No alcohol!" will be tested with an RCT with 2 conditions. A total of 656 mothers and their 6 grade children (11-12 years old) will be involved in the experimental group receiving the program, and 656 mothers and their children will participate in the control condition. Mother-child dyads in the control group receive a factsheet brochure on youth alcohol use and the detrimental consequences of alcohol use among children, which is the standard alcohol brochure of the Trimbos Institute (the Netherlands Institute for Mental Health and Addiction). The experimental group receives 5 modules on a monthly basis. After baseline assessment of children and mothers, follow-up assessments will be conducted after 6, 12, and 18 months (see Figure 1). Assessments will be conducted among both children and mothers at each time point.

At the end of the project, 10 travel checks of 500 euro's will be raffled between families who filled in the questionnaire at each time point. Children will receive a small gift to thank them for participating in the study.

### Participants

#### Recruitment

Respondents will be recruited through a selected sample of primary schools in the Netherlands. Principals of participating primary schools are asked to hand out envelopes for the mothers to children from grade 6 of Dutch elementary school, who are 11-12 years old at the time the prevention starts. This envelop includes a letter in which we ask mothers to participate with their children in a study testing an alcohol prevention program, an informed consent form for themselves and their children, and a response envelop. If mothers and their children want to participate, they can return their contact information by means of the informed consent form in the enclosed response envelop. Also, mothers and children can read information about the study and register online via a webpage.

#### Inclusion criteria

To be included in the present study, children will have to be in grade 6 of Dutch elementary school, when most children are 11-12 years old. Children can only participate together with their mother or a female guardian, and they both have to be able to speak and read Dutch. The form with which mothers and children sign up for the study also serves as an informed consent form. The proposed study and prevention protocols have been approved by the ethical committee of the Faculty of Social Sciences at the Radboud University Nijmegen (ECG16092010).

We focus on grade 6 children (11-12 years old) at baseline, because children start to get increasingly interested in alcohol issues at this age [18], but have generally not drunk their first glass of alcohol yet [19], which makes them an important target group for primary prevention. Providing an alcohol prevention program just before the age of onset, might have a large impact on the health of these children [8]. Inclusion of younger children might not be appropriate, because they will be less likely to consider drinking alcohol themselves. Thus, the period will be too long before they think of trying alcohol. Further, this age group consists of children who are in late childhood, prior to early adolescence. Early adolescence is characterized by increased conflicts with parents, especially with mothers [20] leading to less conformity and openness. This pleads for a focus on the developmental period prior to early adolescence - late childhood - when children are expected to be still quite susceptible to the influence of their parents [21]. While Dutch prevalence figures indicate that 13 to 17% of the 11 years old children drank at least one glass of alcohol [19,22] and we expect this also to be the case in our sample at baseline, we expect that the prevention program "In control: No alcohol!" will significantly lower the increase of this percentage a year later.

We have a few reasons to focus on mothers as target parents: (a) most children spend more time with their mother than with their father, giving mothers the practical advantage of having more time to deliver the alcohol-specific socialization program to their children [23], (b) if parents are divorced, in most cases children live with their mothers [23], (c) women generally are more likely than men to enroll in health-related programs [24], (d) the smoking-specific program also included only mothers, and (e) given the plausibility that program effects would differ by parent gender, including fathers would substantially increase the size and costs of the proposed trial. However, since fathers drink more alcohol than mothers [23], we measure paternal drinking behaviors in the questionnaires of both mothers and children, to be able to control for paternal drinking in the analyses.

#### Randomization

Randomization will take place at the school level, to avoid contamination between conditions. This means that all children in one school will be allocated to the same condition, prevention or control. An independent statistician will perform the allocation of schools to the two conditions.

### Sample Size Calculation

Based on the outcomes of parent-adolescent interventions in the Netherlands and the United States [14,21], we expect a minimal 10% difference in initiation rates between the control and experimental group at the third follow up, which is approximately 12 months after the end of the prevention. Equal cell sizes are assumed for study cells and power of .80 has been targeted. The primary hypothesis, a significant lower percentage of children who have their first glass of alcohol in the prevention group than in the control group, will be tested at an overall significance level of 0.05 (two-sided). G-Power was used to calculate the estimated sample sizes for two-sample comparison of proportions. Based on the prevalence of alcohol use in 13 year olds (age of the children at 18 months follow-up), which is 55%, we need 404 children per condition. However, if we take into account possible attrition (0.80), the fact that data are clustered (mother-child dyads are nested within schools) and the fact that we apply multiple imputation in the case of missing data (factor 1.4), we end up with 656 children per condition ((183/0.80) * 1.4)). Thus, 1312 mothers (and children) are required to participate. In accordance with the intention-to-treat philosophy, all children randomized to one of the conditions are included in analyses to test the study hypotheses.

### Program

#### Theoretical basis of the program

The program was structured around two theories to meet the prevention objectives: Social Cognitive Theory and models of persuasive communication for attitude and behavior change. Fundamentals of child socialization were derived from Bandura's Social Cognitive Theory [4] and consisted of perception (the articulated thoughts and actions of parents or other socializing agents are noticed by the child); cognitive rehearsal (recall and assignment of meaning to what has been noticed by the child); behavioral rehearsal (rehearsal of the things learned while receiving feedback regarding these thoughts and behaviors); and motivation (reinforcement for certain communications and actions). Every part of the program addresses one or more of these child socialization processes.

The Elaboration Likelihood Model [25] contributed to the design of persuasive communication. This model states that participants can differ in the degree to which they experience the program being relevant or obvious. While designing the prevention content and layout, this should be taken into account. For example, some parents will respond to program recommendations most through message content while others' may be most affected by peripheral cues such as print design. Both content and layout are taken into account while structuring program information.

Other program design strategies which are theory-based and have been used to develop the alcohol-specific socialization program include: (a) allow participating parents to exercise choice regarding when and how to implement program objectives, which will increase the probability that for example alcohol using parents will participate, (b) begin with "small wins" that are easy to achieve and build parental confidence, and thereafter, promote gradual change in socialization activities, (c) dedicate part of the prevention to developing the requisite skills, such as parent-child communication skills, needed to implement other program recommendations, (e) build program recommendations on alcohol-specific socialization literature, and (d) use multiple reinforcers, including self-monitoring and feedback (and a small financial incentive at the end) to maintain involvement and motivation.

#### Prevention condition

The proposed program, "In control: No alcohol!", consists of 5 modules which families receive by mail every 4 weeks for a period of 5 months. A module consists of an attractive magazine including information, games, quizzes, and puzzles for parents and children to complete together. These structured interactions for the parent and child is a key technique for facilitating parent-child engagement in the program. The magazines' content is based on the empirical evidence of alcohol-specific parenting in the delay of early alcohol intake (e.g., [7-9,26]).

Each of the five magazines addresses different important issues regarding youth alcohol use and child socialization. Magazine 1 consists of general information about alcohol, alcohol use among children and the importance of parenting behavior, such as anti-alcohol norms and parental supervision. Magazine 2 addresses the risks of alcohol use, especially among children, and parental attitudes towards early drinking. Magazine 3 focuses on parental modeling of alcohol use and the effectiveness of setting rules about alcohol, also for parents who use alcohol themselves. Magazine 4 aims at enhancing awareness about peer influence and increasing the ability to handle peer pressure, while magazine 5 discusses the influence of alcohol-related media and again stressed the eminence of setting clear and strict rules. In addition to these specific topics, each magazine contains general information and practical tips on high-quality parent-child communication in order to gradually increase parents' skill and comfort level in communicating with their children about alcohol.

In addition, with the first magazine the child receives a personalized activity book ("Logboek"). The activity book provides the child the opportunity to repeat what he/she learned about alcohol in a playful and personal way. It is also an extra stimulus to be active in the program. With the activity book, each child receives a personal login code for the related secured website http://www.houvolgeenalcohol.nl. The login code provides access to more games, puzzles and pictures related to the prevention program. The child can download the completed website activities and put them in his/her activity book, so he/she can create his/her own glossy journal.

#### Control condition

Mother-child dyads in the control group receive a factsheet information brochure on youth alcohol use and the detrimental consequences of alcohol use among children, which is the standard alcohol brochure of the Trimbos Institute. Providing a brochure for controls was done primarily to establish a plausible explanation regarding the need for the post-treatment survey for participants in the control condition. The brochure will give them the idea that they are participating in an alcohol prevention study. We choose for this brochure, because it is already available for all Dutch parents, and can be found in several health institutions. It is easily accessible for parents who have an interest in youth alcohol use, so basically many Dutch parents were already exposed to this type of prevention. Although this information could increase mothers' knowledge regarding alcohol issues, this knowledge is not expected to have an effect on alcohol-specific socialization processes or on children's susceptibility to or initiation of alcohol use, since it will not include any tools for mothers on how to use alcohol-specific parenting.

### Data Collection

Both mothers and children will receive separate personal login codes by email. With these login codes they have access to their own baseline questionnaire on a secured webpage. In the case a mother or child prefers a paper questionnaire, this will be sent to their home. Mothers and children are explicitly asked to fill in the questionnaires separately. This questionnaire procedure will take place at each assessment. Non-responding mothers or children will be approached by phone to motivate them to fill in the questionnaire. Mothers and children in both the experimental and control condition will be assessed at baseline (1 month before prevention starts), after 6 months (first follow up), after 12 months (second follow-up) and after 18 months (third follow up). An overview of all measures at each time point is provided in Table 1.

**Table 1:** Overview of Measures

Measure	Baseline		Follow-up 1		Follow-up 2		Follow-up 3	
	Mother	Child	Mother	Child	Mother	Child	Mother	Child
Demographic characteristics	X	X						
Monitoring	X	X	X	X	X	X	X	X
Parent-child relationship (NRI)		X		X		X		X
Intention to drink alcohol		X		X		X		X
Self-efficacy		X		X		X		X
Drinking norms		X		X		X		X
Alcohol use parents	X	X	X	X	X	X	X	X
Problem drinking parents	X		X		X		X	
Alcohol use child	X	X	X	X	X	X	X	X
Alcohol use peers & siblings		X		X		X		X
Attitude about alcohol	X	X	X	X	X	X	X	X
Alcohol-related consequences	X	X	X	X	X	X	X	X
Anti alcohol socialization								
Availability of alcohol at home	X	X	X	X	X	X	X	X
Rules on alcohol	X	X	X	X	X	X	X	X
Communication about alcohol	X	X	X	X	X	X	X	X
Parental norms	X		X		X		X	
Parental influence on offspring alcohol use	X		X		X		X	
Strengths and Difficulties Questionnaire (SDQ)	X	X	X	X	X	X	X	X
Program evaluation/utilization			X	X				

#### Outcomes

The primary outcome, initiation of alcohol use, is defined as have been drinking at least one glass of alcohol. Secondary outcome measures are alcohol-specific parenting dimensions such as rules about alcohol, non-drinking agreement, alcohol availability at home, and frequency and quality of alcohol-specific communication (e.g., [7,26]), but also general parental monitoring [27] and parent-child relationship quality [28]. Other outcomes are susceptibility to drinking alcohol, defined as the lack of a firm commitment against drinking alcohol [29,30], alcohol expectancies [31], self-efficacy [32,33], and frequency and intensity of child alcohol use [34]. The Strengths and Difficulties Questionnaire (SDQ) [35] will be used as a behavioral screening instrument for early detection of psychological problems. Psychological problems are associated with problem behaviors like drinking alcohol at an early age (e.g., [36]).

#### Statistical analyses

In accordance with the intent-to-treat philosophy, all children randomized to a condition will be included in the analyses to test the study hypotheses. Moreover, while randomization takes place on school level and children are 'nested' within these schools, we need to control for clustered data [37]. Mplus is a statistical modeling program that has special features to deal with missing data and it allows analyses with complex data while taking into consideration the longitudinal character of the data and the fact that data are clustered. Regression analyses for dichotomous outcome measures (logistic regression) will be conducted to test whether children in the control condition are more likely to initiate drinking than children in the experimental condition [14,37]. For the second aim of our study, namely, that mothers in the prevention group will use more alcohol-specific socialization strategies than mothers of the control group, we will perform mediation analyses in Mplus, using the bootstrap method [37]. To test our third hypothesis of the study, possible moderating effects of relevant demographic indices such as gender, as well as mothers' alcohol use at baseline, we will create products of the predictors and then include those interaction terms in the logistic regression model (e.g., condition * mothers' drinking; [37]).

## Discussion

The present study protocol presents the design of an RCT evaluating the effectiveness of the "In control: No alcohol!" program for 6 grade children. This universal prevention program aims to delay the age of alcohol onset for Dutch children. It is hypothesized that mothers in the prevention condition will employ more alcohol socialization practices, and that children in the prevention condition will be less likely to have their first glass of alcohol, compared to the control condition.

### Strengths and limitations

An important strength of the program "In control: No alcohol!" is that it is theory driven. The underlying structure of the program is based on the Social Cognitive Learning Theory [4], and the Elaboration Likelihood Model [25], while the content is based on recent alcohol-specific parenting research [7-10,26]. Second, the program reaches children during the pre-initiation stage of alcohol use, aiming to prevent them from drinking their first alcoholic beverage, and thereby lowering the odds of heavy drinking [2,3]. Third, parents can complete the program with their children at home at a time of their choice. This creates the opportunity to include parents in the study, who are normally more difficult to reach for alcohol prevention, like parents who drink alcohol.

A strength of the study design is that it also includes long-term follow-ups at 12 and 18 months, in addition to the immediate follow-up at 6 months. This will create more opportunity to find an effect on actual alcohol use of the children, as well as mediating effects through alcohol-specific parenting practices. Further, if the "In control: No alcohol!" program turns out to be effective, it can easily be implemented on a large scale via primary schools. A limitation of the study is that only mothers can participate. While there are several good reasons for this choice (e.g. mothers are more likely to spend time with their children and to enroll in health-related programs), previous research has shown differences in alcohol-specific socialization between mothers and fathers; For example, mothers communicate more often about alcohol [7,38] and are more understanding towards their children [39] compared to fathers. In future research, the effect of the program, when targeted at fathers, should be investigated.

### Implications for practice

If the "In control: No alcohol!" program turns out to be effective, it can be implemented on a large scale in a reasonable amount of time. The program's modular, self-help format allows flexibility as regards where, when, and how it is implemented. Although the proposed study will measure effects on individual children after delivering the modules to households, in the future, the program could also be self-administered on a website that provides sequential access to the prevention modules. This is one of the main reasons that the Trimbos Institute (the Netherlands Institute for Mental Health and Addiction) is actively involved in this project. Collaboration with the Trimbos Institute guarantees that the program will be widespread and will reach large populations. Another advantage of the close collaboration with Trimbos Institute is that the results of this study can be transferred to practice immediately hardly without delay.

## Conclusion

This study will evaluate a protocol for preventing early alcohol onset in children. The results of this study will provide insights into the effectiveness of the "In control: No alcohol!" prevention program and the antecedents of alcohol use among children.

## Competing interests

Jacqueline Verdurmen and Ingrid Schulten work for the Trimbos Institute, which is the institute that co-developed the "In control: No alcohol!" program, together with Haske van der Vorst and Rutger Engels. The other authors declare that they have no competing interests.

## Authors' contributions

SM is responsible for the data collection, data analysis and for reporting the study results. All other authors are supervisors and grant applicators. All authors read and approved the final manuscript.

## Pre-publication history

The pre-publication history for this paper can be accessed here:

http://www.biomedcentral.com/1471-2458/11/622/prepub
